# Supplementary material for: Mechanistic aspects of maltotriose-conjugate translocation to the Gram-negative bacteria cytoplasm
Source: Life Sci Alliance. 2018 Dec 28;2(1):e201800242. doi: 10.26508/lsa.201800242 (PMC6311466; doi:10.26508/lsa.201800242)
Supplement: Supplementary file 1 [file LSA-2018-00242_Supplementary_Materials_and_Methods.docx]

# Supplementary Materials and Methods

## **Chemicals (synthesis, structure)**

Cpd-2 was prepared as described in Ning *et al.*^a^ (MDP-1 compound). The preparation of Cpd-1 follows a similar route and is show below.

**Synthesis of *O*-(α-*D*-glucopyranosyl)-(1→4)-(α-*D*-glucopyranosyl)-(1→4)-β-*D*-glucopyranosyl-(1→1)-1-(3′-triazolepropyl perylene) (2*R*,3*S*,4*S*,5*S*,6*R*)-2-(((2*R*,3*S*,4*R*,5*S*,6*R*)-6-(((2*R*,3*S*,4*R*,5*S*,6*R*)-4,5-dihydroxy-2-(hydroxymethyl)-6-(3-(4-((perylen-3-ylmethoxy)methyl)-1*H*-1,2,3-triazol-1-yl)propoxy)tetrahydro-2*H*-pyran-3-yl)oxy)-4,5-dihydroxy-2-(hydroxymethyl)tetrahydro-2*H*-pyran-3-yl)oxy)-6-(hydroxymethyl)tetrahydro-2*H*-pyran-3,4,5-triol (1)**.

**(3*S*,4*S*,5*R*,6*R*)-6-(Acetoxymethyl)-5-(((2*R*,3*S*,4*S*,5*R*,6*R*)-3,4-diacetoxy-6-(acetoxymethyl)-5-(((2*R*,3*S*,4*S*,5*R*,6*R*)-3,4,5-triacetoxy-6-(acetoxymethyl)tetrahydro-2*H*-pyran-2-yl)oxy)tetrahydro-2*H*-pyran-2-yl)oxy)tetrahydro-2*H*-pyran-2,3,4-triyl triacetate (3).** An oven-dried round-bottom flask (100 mL) was cooled under a stream of argon and charged with maltotriose (4.96 mmol, 2.50 g, 1 equiv) and DMAP (9.91 mmol, 1.21 g, 2 equiv). Anhydrous pyridine (40 mL) was added and the resulting yellowish solution was cooled to 2-4 ^o^C (crushed ice). Acetic anhydride (148.68 mmol, 15.18 g, 14.07 mL, 30 equiv) was added dropwise within 15 min, and the yellow solution was stirred at room temperature for 18 h under atmosphere of argon. The progress of the reaction was monitored by TLC (2:1 EtOAc/hexanes; visualization of TLC spots required immersing the TLC plate into a solution of sulfuric acid in ethanol (1:15 v:v) and subsequent drying the TLC plate with heat-gun). Upon complete conversion of the starting maltotriose, the yellow reaction mixture was concentrated under vacuum, the brown residue was diluted with EtOAc (100 mL) and washed with aqueous saturated NaHCO_3_ solution (50 mL) and then by water (100 mL). The aqueous layer was back-extracted with EtOAc (3x100 mL). Combined organic extracts were dried over Na_2_SO_4_, filtered and evaporated to dryness under vacuum. The residue was purified by column chromatography (Biotage SNAP KP-Sil 100 g; gradient elution from 25% EtOAc in petroleum ether to 100% EtOAc) to afford **3** as a white foam (3.95 g, 82%); analytical TLC on silica gel, 2:1 EtOAc/hexanes, R*_f_* = 0.52 (staining of TLC spots with the solution of sulfuric acid in ethanol (1:15 v:v) was used; see above). ^1^H NMR (CDCl3, 400 MHz,) *δ* 6.23 (0.5H, d, J=3.7 Hz, α-H) 5.73 (0.5H, d, J=8.1 Hz, β-H) 5.50 (1H, dd, J=10.1, 8.8 Hz) 5.42-5.39 (1H, m) 5.39-5.35 (1H, m) 5.34-5.30 (1H, m) 5.29-5.24 (1H, m) 5.05 (1H, td, J=10.1, 2.6 Hz) 4.97-4.92 (1H, m) 4.84 (1H, dd, J=10.5, 4.0 Hz) 4.73 (1H, dt, J=10.5, 4.0 Hz) 4.48-4.41 (2H, m) 4.32-4.26 (1H, m) 4.26-4.21 (1H, m) 4.18-4.13 (1H, m) 4.13-4.07 (1H, m) 4.06-4.02 (1H, m) 4.01-3.97 (1H, m) 3.96-3.92 (2H, m) 3.90-3.83 (1H, m) 2.22 (1.5H, s) 2.16 (1.5H, s) 2.15 (3H, s) 2.14-2.13 (3H, m) 2.09-2.08 (3H, m) 2.04 (3H, s) 2.02-2.01 (3H, m) 2.01-2.00 (3H, m) 1.99 (3H, s) 1.98 (3H, s) 1.98-1.97 (3H, m) 1.97 (3H, s). The ^1^H NMR spectrum was in agreement with that reported in the literature for a 1:1 mixture of α/β-anomers^b^. ^13^C NMR (101 MHz, CDCl3) *δ* 170.8, 170.7, 170.7, 170.7, 170.6, 170.6, 170.6, 170.6, 170.4, 170.4, 170.1, 170.0, 170.0, 169.9, 169.9, 169.9, 169.7, 169.6, 169.5, 169.1, 168.9, 96.1, 96.0, 95.8, 95.7, 91.4, 89.0, 77.2, 75.3, 73.6, 73.4, 73.1, 72.6, 72.4, 72.3, 71.9, 71.8, 71.1, 70.6, 70.6, 70.3, 70.2, 70.2, 69.8, 69.5, 69.2, 68.6, 68.0, 62.8, 62.7, 62.4, 62.3, 61.5, 61.5, 21.2, 21.1, 21.0, 21.0, 20.9, 20.8, 20.7, 20.7, 20.7, 20.6; HRMS-ESI (m/z) calcd for C_40_H_54_O_27_Na [M+Na]^+^ 989.2750, found 989.2750.

**(2*R*,3*R*,4*S*,5*S*,6*R*)-2-(Acetoxymethyl)-6-(((2*R*,3*R*,4*S*,5*S*,6*R*)-4,5-diacetoxy-2-(acetoxymethyl)-6-(((2*R*,3*R*,4*S*,5*S*)-4,5-diacetoxy-2-(acetoxymethyl)-6-hydroxytetrahydro-2*H*-pyran-3-yl)oxy)tetrahydro-2*H*-pyran-3-yl)oxy)tetrahydro-2*H*-pyran-3,4,5-triyl triacetate (4).** Hydrazine acetate (4.84 mmol, 445 mg, 1.25 equiv) was added to a solution of peracetylated maltotriose **3** (3.87 mmol, 3.74 g, 1 equiv) in anhydrous DMF (40 mL) under atmosphere of argon and the resulting colorless solution was heated at 60 ºC for 18 hours. Progress of the reaction was monitored by TLC (2:1 EtOAc/hexanes; visualization of TLC spots required immersing the TLC plate into a solution of sulfuric acid in ethanol (1:15 v:v) and subsequent drying the TLC plate with heat-gun). Upon complete conversion the yellow reaction mixture was concentrated under vacuum. The residue was diluted with EtOAc (100 mL) and washed with water (50 mL), and then with brine (100 mL). The aqueous layer was back-extracted with EtOAc (3x100 mL). Organic layers were combined, dried over Na_2_SO_4_, filtered and concentrated to dryness under vacuum. The residue was purified by column chromatography (Biotage SNAP KP-Sil 100 g; gradient elution from 25% EtOAc in petroleum ether to 100% EtOAc) to afford **3** as a colorless oil. The oil was dissolved in dichloroethane and all volatiles were removed in vacuo. The dissolution/evaporation sequence was repeated two more times to afford the desired product **4** as a white foam (2.75 g, 77%); analytical TLC on silica gel, 2:1 EtOAc/hexanes, R*_f_* = 0.32 (staining of TLC spots with the solution of sulfuric acid in ethanol (1:15 v:v) was used; see above). ^1^H NMR (400 MHz, CDCl_3_) *δ* 5.57 (0.6H, dd, J=10.0, 8.8 Hz) 5.44-5.25 (5.4H, m) 5.09-5.03 (1H, m) 4.87-4.82 (1H, m) 4.79-4.72 (2H, m) 4.52-4.43 (2H, m) 4.34-4.14 (4H, m) 4.08-4.02 (1H, m) 4.00-3.90 (4H, m) 3.80-3.73 (0.4H, m, β–OH) 3.40-3.26 (0.6H, br s, α–OH) 2.18-2.16 (3H, m) 2.14 (3H, s) 2.09 (3H, s) 2.06-2.04 (6H, m) 2.03-2.01 (6H, m) 2.01-1.98 (9H, m). The ^1^H NMR spectrum was in agreement with that reported in the literature (a 3:2 mixture of α:β-anomers)^c^. ^13^C NMR (101 MHz, CDCl3) *δ* 170.9, 170.9, 170.8, 170.7, 170.7, 170.7, 170.5, 170.5, 170.4, 170.1, 170.0, 170.0, 169.8, 169.6, 95.8, 95.8, 95.8, 95.1, 90.2, 77.2, 74.8, 74.0, 73.8, 72.7, 72.6, 72.6, 72.4, 71.9, 71.8, 71.7, 70.6, 70.2, 69.5, 69.2, 69.1, 68.6, 68.1, 67.9, 67.2, 63.2, 63.1, 62.4, 61.5, 21.1, 21.0, 21.0, 21.0, 20.9, 20.8, 20.8, 20.7; HRMS-ESI (m/z) calcd for C_38_H_52_O_26_Na [M+Na]^+^ 947.2645, found 947.2664.

**(*2R,*3*R*,4*S*,5*S*,6*R*)-2-(Acetoxymethyl)-6-(((2*R*,3*R*,4*S*,5*S*,6*R*)-4,5-diacetoxy-2-(acetoxymethyl)-6-(((2*R*,3*R*,4*S*,5*S*,6*R*)-4,5-diacetoxy-2-(acetoxymethyl)-6-(2,2,2-trichloro-1-iminoethoxy)tetrahydro-2*H*-pyran-3-yl)oxy)tetrahydro-2*H*-pyran-3-yl)oxy)tetrahydro-2*H*-pyran-3,4,5-triyl triacetate (5).** Anhydrous K_2_CO_3_ (25.95 mmol, 3.59 g, 10 equiv) was added to a colorless solution of maltotriose **4** (2.60 mmol, 2.40 g, 1 equiv) and trichloroacetonitrile (25.95 mmol, 3.75 g, 2.60 mL, 10 equiv) in anhydrous CH_2_Cl_2_ (50 mL). The resulting yellowish suspension was stirred at ambient temperature for 18 h, and progress of the reaction was monitored by TLC (2:1 EtOAc/hexanes; visualization of TLC spots required immersing the TLC plate into a solution of sulfuric acid in ethanol (1:15 v:v) and subsequent drying the plate with heat-gun). Upon complete conversion of the starting maltotriose **3**, the yellow suspension was diluted with water (200 mL). Layers were separated and the aqueous layer was back-extracted with CH_2_Cl_2_ (3x100 mL). Combined organic layers were dried over Na_2_SO_4_, filtered and concentrated under vacuum. The yellow oily residue was purified by column chromatography (Biotage SNAP KP-Sil 100 g; gradient elution from 25% EtOAc/petroleum ether to 100% EtOAc) to afford the target product **5** as a colorless oil. The oil was dissolved in dichloroethane and all volatiles were removed in vacuo. The dissolution/evaporation sequence was repeated two more times to afford the desired product **5** as a white foam (2.25 g, 81%); analytical TLC on silica gel, 2:1 EtOAc/hexanes, R*_f_* = 0.57 (staining of TLC spots with the solution of sulfuric acid in ethanol (1:15 v:v) was used; see above). ^1^H NMR (400 MHz, CDCl_3_) *δ* 8.67 (1H, s) 6.48 (1H, d, J=3.8 Hz) 5.59 (1H, dd, J=10.0, 8.8 Hz) 5.44-5.39 (2H, m) 5.35 (1H, dd, J=10.5, 9.5 Hz) 5.30 (1H, d, J=4.0 Hz) 5.07 (1H, dd, J=10.2, 9.5 Hz) 5.02 (1H, dd, J=9.8, 3.8 Hz) 4.85 (1H, dd, J=10.5, 4.0 Hz) 4.76 (1H, dd, J=10.2, 4.0 Hz) 4.54-4.46 (2H, m) 4.31-4.23 (2H, m) 4.22-4.14 (2H, m) 4.08-4.00 (2H, m) 3.97-3.90 (3H, m) 2.16 (3H, s) 2.15 (3H, s) 2.10 (3H, s) 2.06 (3H, s) 2.04 (3H, s) 2.03 (3H, s) 2.02-2.01 (6H, m) 1.99 (3H, s) 1.98 (3H, s); ^13^C NMR (101 MHz, CDCl3) *δ* 170.8, 170.7, 170.6, 170.5, 170.4, 170.1, 169.9, 169.8, 169.7, 169.6, 161.1, 96.2, 95.8, 92.9, 90.9, 77.2, 73.5, 72.6, 71.9, 71.9, 70.7, 70.6, 70.2, 69.5, 69.2, 68.6, 68.0, 62.5, 62.3, 61.5, 21.1, 21.0, 21.0, 20.9, 20.8, 20.7, 20.5; IR (film, cm^−1^) 1748 (C=O) 1234 (C-O-C) 1035 (C-O-C); [α]^20^_D_+109.8 (*c* 1.01, CHCl_3_); HRMS-ESI (m/z) calcd for C_40_H_52_NO_26_NaCl_3_ [M+Na]^+^ 1090.1741, found 1090.1760.

**(2*R*,3*R*,4*S*,5*S*,6*R*)-2-(Acetoxymethyl)-6-(((2*R*,3*R*,4*S*,5*S*,6*R*)-4,5-diacetoxy-2-(acetoxymethyl)-6-(((*2R*,3*R*,4*S*,5*S*,6*R*)-4,5-diacetoxy-2-(acetoxymethyl)-6-(3-azidopropoxy)tetrahydro-2*H*-pyran-3-yl)oxy)tetrahydro-2*H*-pyran-3-yl)oxy)tetrahydro-2*H*-pyran-3,4,5-triyl triacetate (6)**. 4Å Molecular sieves (1 g) were added to a colorless solution of trichloroacetimidate **5** (0.94 mmol, 1.0 g, 1 equiv) and 3-azido-1-propanol (3.74 mmol, 380 mg, 345 µl, 4 equiv) in anhydrous CH_2_Cl_2_ (30 mL). The resulting suspension was cooled to 2–4 ^o^C (crushed ice) and TMSOTf (0.25 M solution in CH_2_Cl_2_, 1.17 mmol, 260 mg, 212 µl, 1.25 equiv) was added dropwise within 30 min. The colorless solution was stirred at 2–4^o^C for 2 h, and progress of the reaction was monitored by TLC (2:1 EtOAc/hexanes; visualization of TLC spots required immersing the TLC plate into a solution of sulfuric acid in ethanol (1:15 v:v). Upon complete conversion of the starting trichloroacetimidate **5**, the reaction mixture was quenched with NEt_3_ (2.34 mmol, 237 mg, 325 µl, 2.5 equiv) at 2–4^o^C and warmed to room temperature within 10 min. Water (150 mL) was then added and layers were separated. The aqueous layer was back-extracted with CH_2_Cl_2_ (3x50 mL). Combined organic layers were dried over Na_2_SO_4_, filtered and concentrated in vacuo. The residue was purified by column chromatography (Biotage SNAP KP-Sil 50 g; gradient elution from 10% EtOAc in petroleum ether to 100% EtOAc) to afford the desired azide **6** as a white foam (375 mg, 39%); analytical TLC on silica gel, R*_f_* = 0.65, 2:1 EtOAc/hexanes (staining of TLC spots with the solution of sulfuric acid in ethanol (1:15 v:v) was used; see above). ^1^H NMR (400 MHz, CDCl_3_) *δ* 5.41-5.31 (3H, m) 5.27-5.22 (2H, m) 5.06 (1H, dd, J=10.2, 9.5 Hz) 4.85 (1H, dd, J=10.5, 4.1 Hz) 4.80 (1H, dd, J=9.5, 7.9 Hz) 4.73 (1H, dd, J=10.5, 4.1 Hz) 4.52 (1H, d, J=7.9 Hz) 4.46 (2H, ddd, J=11.0, 8.9, 2.5 Hz) 4.30 (1H, dd, J=12.0, 4.2 Hz) 4.24 (1H, dd, J=12.4, 3.6 Hz) 4.17 (1H, dd, J=12.4, 3.6 Hz) 4.04 (1H, dd, J=12.5, 2.5 Hz) 4.00-3.89 (5H, m) 3.73-3.68 (1H, m) 3.62-3.56 (1H, m) 3.41-3.31 (2H, m) 2.16 (3H, s) 2.14 (3H, s) 2.09 (3H, s) 2.04 (3H, s) 2.02 (3H, s) 2.02-2.00 (6H, m) 2.00-1.98 (6H, m) 1.98 (3H, s) 1.87-1.78 (2H, m); ^13^C NMR (101 MHz, CDCl3) *δ* 170.7, 170.7, 170.7, 170.6, 170.5, 170.2, 170.0, 169.8, 169.8, 169.6, 100.4, 95.9, 95.8, 77.2, 75.4, 73.9, 72.6, 72.3, 72.3, 71.9, 70.6, 70.2, 69.5, 69.1, 68.6, 68.0, 66.6, 63.0, 62.5, 61.5, 48.1, 29.1, 21.0, 21.0, 20.9, 20.8, 20.8, 20.7, 20.7, 20.7; IR (film, cm^−1^) 2101 (N3) 1756 (C=O) 1234 (C-O-C) 1039 (C-O-C); [α]^20^_D_+76.9 (*c* 1.64, CHCl_3_); HRMS-ESI (m/z) calcd for C_41_H_57_N_3_O_26_Na [M+Na]^+^ 1030.3128, found 1030.3123.

**(2*R*,3*R*,4*S*,5*S*,6*R*)-2-(Acetoxymethyl)-6-(((2*R*,3*R*,4*S*,5*S*,6*R*)-4,5-diacetoxy-2-(acetoxymethyl)-6-(((2*R*,3*R*,4*S*,5*S*,6*R*)-4,5-diacetoxy-2-(acetoxymethyl)-6-(3-(4-((perylen-3-ylmethoxy)methyl)-1*H*-1,2,3-triazol-1-yl)propoxy)tetrahydro-2*H*-pyran-3-yl)oxy)tetrahydro-2*H*-pyran-3-yl)oxy)tetrahydro-2H-pyran-3,4,5-triyl triacetate (8)**. To a mixture of azide **5** (0.298 mmol, 300 mg, 1 equiv), 3-prop-2-ynyloxymethyl-perylene **7** (0.595 mmol, 191 mg, 2 equiv) and CuI (0.030 mmol, 6 mg, 10 mol%, 0.1 equiv) in an oven-dried pressure vial (20 mL) was added anhydrous DMF (10 mL) and DIPEA (0.595 mmol, 77 mg, 106 µl, 2 equiv). The resulting yellow solution was stirred at room temperature for 18 hours under atmosphere of argon, and progress of the reaction was monitored by TLC (2:1 EtOAc/hexanes; visualization of TLC spots required immersing the TLC plate into a solution of sulfuric acid in ethanol (1:15 v:v). Upon complete conversion of the starting azide **6**, the yellow reaction mixture was concentrated under vacuum. The yellowish oily residue was diluted with EtOAc (50 mL) and washed with brine (100 mL). The aqueous layer was back-extracted with EtOAc (3x50 mL). Combined organic layers were dried over Na_2_SO_4_, filtered and evaporated to dryness under vacuum. The residue was purified by column chromatography (Biotage SNAP KP-Sil 25 g; gradient elution from 25% EtOAc/petroleum ether to 100% EtOAc) to afford triazole **8** as a yellow foam (310 mg, 78%); analytical TLC on silica gel, 1:1 EtOAc/hexanes, R*_f_* = 0.30. ^1^H NMR (400 MHz, CDCl_3_) *δ* 8.25-8.12 (4H, m) 7.95-7.91 (1H, m) 7.71-7.66 (2H, m) 7.56-7.45 (5H, m) 5.44-5.39 (2H, m) 5.37 (1H, dd, J=10.5, 9.4 Hz) 5.26 (1H, d, J=4.1 Hz) 5.21 (1H, dd, J=9.4, 8.8 Hz) 5.07 (1H, dd, J=10.2, 9.4 Hz) 5.00 (2H, s) 4.86 (1H, dd, J=10.5, 4.1 Hz) 4.81-4.71 (4H, m) 4.49-4.31 (5H, m) 4.28-4.14 (3H, m) 4.05 (1H, dd, J=12.5, 2.4 Hz) 3.98-3.89 (4H, m) 3.79-3.71 (1H, m) 3.61-3.55 (1H, m) 3.44-3.36 (1H, m) 2.14 (3H, s) 2.14 (3H, s) 2.13-2.07 (5H, m) 2.05 (3H, s) 2.04-2.02 (6H, m) 2.01-2.00 (6H, m) 2.00 (3H, s) 1.98 (3H, s); ^13^C NMR (101 MHz, CDCl3) *δ* 170.8, 170.7, 170.7, 170.5, 170.5, 170.2, 170.0, 169.9, 169.9, 169.6, 145.3, 134.8, 133.2, 133.2, 131.7, 131.7, 131.3, 131.2, 129.1, 128.6, 128.1, 128.1, 127.7, 127.0, 126.8, 126.7, 124.1, 123.1, 120.6, 120.5, 119.7, 100.3, 95.9, 95.8, 77.2, 75.3, 73.7, 72.6, 72.3, 72.2, 71.9, 71.3, 70.6, 70.2, 69.5, 69.1, 68.6, 68.0, 66.0, 64.0, 62.8, 62.4, 61.5, 46.8, 30.3, 21.1, 21.0, 20.9, 20.8, 20.8, 20.7, 20.7; IR (film, cm^−1^) 1755 (C=O) 1236 (C-O-C) 1043 (C-O-C); [α]^20^_D_+57.7 (*c* 0.51, CHCl_3_) HRMS-ESI (m/z) calcd for C_65_H_74_N_3_O_27_ [M+H]^+^ 1328.4510, found 1328,4524.

**(2*R*,3*S*,4*S*,5*S*,6*R*)-2-(((2*R*,3*S*,4*R*,5*S*,6*R*)-6-(((2*R*,3*S*,4*R*,5*S*,6*R*)-4,5-Dihydroxy-2-(hydroxymethyl)-6-(3-(4-((perylen-3-ylmethoxy)methyl)-1*H*-1,2,3-triazol-1-yl)propoxy)tetrahydro-2*H*-pyran-3-yl)oxy)-4,5-dihydroxy-2-(hydroxymethyl)tetrahydro-2*H*-pyran-3-yl)oxy)-6-(hydroxymethyl)tetrahydro-2*H*-pyran-3,4,5-triol (1)**. Aqueous lithium hydroxide (1M solution in water, 7.00 mmol, 294 mg, 30 equiv) was added to a solution of triazole **8** (0.233 mmol, 310 mg, 1 equiv) in MeOH (30 mL), and the resulting yellow solution was stirred for 18 hours at room temperature under atmosphere of argon. All volatiles were removed in vacuo and the crude product was purified by reversed–phase column chromatography (Biotage SNAP KP-C18-HS 30 g; gradient elution from 100% water to 80% MeCN in water) to afford the desired **1** as a yellow powder (145 mg, 68%). ^1^H NMR (400 MHz, DMSO-d*_6_*) *δ* 8.41-8.30 (4H, m) 8.18 (1H, s) 7.92-7.88 (1H, m) 7.82-7.77 (2H, m) 7.61-7.52 (4H, m) 5.62-5.54 (2H, m) 5.53-5.46 (2H, m) 5.24 (1H, d, J=4.9 Hz) 5.02 (2H, dd, J=11.0, 3.8 Hz) 4.93 (2H, s) 4.91-4.87 (2H, m) 4.69 (2H, s) 4.57-4.44 (5H, m) 4.17 (1H, d, J=7.7 Hz) 3.80-3.73 (1H, m) 3.72-3.54 (7H, m) 3.53-3.36 (6H, m) 3.38-3.34 (2H, m, overlapped with water) 3.27-3.20 (2H, m) 3.11-3.02 (2H, m) 2.11-2.04 (2H, m); ^13^C NMR (101 MHz, CDCl3) *δ* 143.7, 134.2, 133.6, 132.5, 130.8, 130.5, 130.5, 130.3, 128.1, 128.0, 127.6, 127.3, 127.0, 126.9, 124.3, 124.1, 120.8, 120.8, 120.7, 120.1, 102.7, 100.8, 100.6, 79.8, 79.5, 79.2, 76.2, 75.1, 73.5, 73.3, 73.2, 73.0, 72.6, 72.0, 71.7, 69.9, 69.6, 65.4, 63.0, 60.8, 60.5, 60.3, 46.4, 39.5, 30.0; IR (film, cm^−1^) 3434 (CH-O-H) 1027 (C-O-C); [α]^20^_D_+51.5 (*c* 0.98, DMSO); HRMS-ESI (m/z) calcd for C_45_H_54_N_3_O_17_ [M+H]^+^ 908.3453, found 908.3447.

a. Ning X, Lee S, Wang Z, Kim D, Stubblefield B, Gilbert E, Murthy N (2011) Maltodextrin-based imaging probes detect bacteria *in vivo* with high sensitivity and specificity. *Nat Mater* **10**: 602–607.

b. Koto S, Haigoh H, Shichi S, Hirooka M, Nakamura T, Maru C, Fujita M, Goto A, Sato T, Okada M, et al. (1995) Synthesis of glucose-containing linear oligosaccharides having α(1→4) and α(1→6) linkages using stereoselective dehydrative glycosylation. *Bull Chem Soc Jpn* **68**: 2331–2348.

c. Wang R, Chen J-Z, Zheng X-A, Kong R, Gong S-S, Sun Q (2018) Hafnium (IV) triflate as a potent catalyst for selective 1-O-deacetylation of peracetylated saccharides. *Carbohydr Res* **455**: 114–118.
