# Supplementary material for: Mechanistic aspects of maltotriose-conjugate translocation to the Gram-negative bacteria cytoplasm
Source: Life Sci Alliance. 2018 Dec 28;2(1):e201800242. doi: 10.26508/lsa.201800242 (PMC6311466; doi:10.26508/lsa.201800242)
Supplement: Supplementary file 2 [file LSA-2018-00242_TableS1.pdf]

Table

Table S1. Summary of the interaction of Cpd-1 or Cpd-2 with LamB revealed from the ion current fluctuation.

	k_{on} [10^6 $M^{-1}s^{-1}$]		*Residence time τ* [μs]	
	+100 mV	*-100 mV*	*+100 mV*	*-100 mV*
**Maltotriose** cis-side addition	1.8±0.6	3.0±0.8	79±16	103±43
**Cpd-1** cis-side addition	0.8±0.3	1.2±0.5	89±29	121±59
**Maltohexaose** cis-side addition	2.7±1.1	3.0±0.8	928±120	1330±140
**Cpd-2** cis-side addition	10.3±3.2	8.7±2.8	1007±131	997±217
**Maltotriose** trans-side addition	6.3±1.8	4.1±1.7	100±20	81±11
**Cpd-1** trans-side addition	2.6±0.4	3.0±0.9	198±57	153±76
**Maltohexaose** trans-side addition	4.6±1.7	5.9±2.8	540±205	908±330
**Cpd-2** trans-side addition	14.8±6.7	15.3±3.4	278±55	300±59

The association rate (k_{on}) and residence time (τ) are calculated from single channel recording. The experiments have been done in 1 M KCl, 10 mM HEPES, pH 7 by applying +/-100 mV external voltage.
